# Uncovering Female Child Sexual Offenders—Needs and Challenges for Practice and Research

**DOI:** 10.3390/jcm8030401

**Published:** 2019-03-22

**Authors:** Safiye Tozdan, Peer Briken, Arne Dekker

**Affiliations:** Institute for Sex Research and Forensic Psychiatry, University Medical Center Hamburg–Eppendorf, 20251 Hamburg, Germany; briken@uke.de (P.B.); dekker@uke.de (A.D.)

**Keywords:** child sexual abuse, female perpetrator, mother-child incest, gender stereotypes, social taboo

## Abstract

This article provides a short literature overview on female child sexual offenders (FCSO) focusing on the discrepancy between prevalence rates from different sources, characteristics of FCSO and their victims, as well as the societal “culture of denial” surrounding these women. FCSO are a powerful social taboo. Even professionals in the healthcare or justice system were shown to respond inappropriately in cases of child sexual abuse committed by women. As a result, offences of FCSO may be underreported and therefore difficult to research. The lack of scientific data on FSCO lowers the quality of child protection and treatment services. We therefore deem it particularly necessary for professionals in health care to break the social taboo that is FCSO and to further stimulate research on the topic of FCSO. We provide some general implications for professionals in health care systems as well as specific recommendations for researchers. We end with an overall conclusion.

## 1. Introduction

### 1.1. Background

Stereotypically, child sexual abuse implies the image of a male perpetrator sexually abusing a female child. However, due to an expanding research field since the 1980s [1], it is well established scientific knowledge today, that part of all child sexual offences are committed by women [2,3,4,5,6].

Although research data on female child sexual offenders (abbreviated female child sexual offenders (FCSO) in the following References [1,2,5,7,8,9,10,11,12,13,14]) is available and can be used for reviews and meta-analyses, there is still a noticeable gap of information on what is known about FCSO as opposed to male child sexual offenders [15]. Additionally, most of what is known resulted from studies with only small clinical samples of female offenders registered by the criminal system [16]. Consequently, the assessment and treatment of FCSO is insufficient [2].

Irrespective of the perpetrator’s gender, child sexual abuse is an underreported crime [17]. One reason for the low level of knowledge about FCSO could be that FCSO are rarely registered in official statistics and are therefore difficult to reach for clinicians and researchers. One possible explanation for this phenomenon is that child sexual abuse committed by women seems to be a powerful social taboo [18]. Therefore, there is a marked resistance against the disclosure of FCSO [19] even among professionals in the health care and justice system [20]. In order to encourage the disclosure of FCSO, enhance the thematic research, and improve the quality of child protection and prevention, we deem it particularly necessary for clinicians and researchers in the field of sexual health to overcome this taboo.

### 1.2. Aim

This article is not a systematic review but is intended to provide a short narrative literature overview on the discrepancy between prevalence rates based on different sources (official reports vs. victimization surveys) and on the adult FCSO’s characteristics (e.g., average age, socioeconomic status, mental health issues, victims). Secondly, we focus on FCSO as a social taboo that even percolates the health care and justice system. In order to overcome this social taboo, we provide some general implications for professionals in health care systems. In order to foster research activities on FCSO, we give specific recommendations for researchers in the field of sexual medicine.

## 2. Method

We explored the current literature on FCSO and mainly included reviews and studies on FCSO from 2000–2019 examining large and/or representative samples. We focused on data from countries sharing similar cultural and societal backgrounds. We included some additional studies that were published before the year 2000, but had an important impact on this research field and are still frequently cited. We excluded articles in which only juvenile FCSO or general female sexual offenders (with adult victims) were analyzed. Search terms included “female”, “woman”, or “mother” with “sexual child abuse”, “child sexual offending”, or “incest” as well as “social taboo” or “gender stereotypes”. Searches were performed in PsychInfo, PubMed, KrimDok, and socINDEX. When necessary, additional references were used (e.g., Google).

## 3. Female Sexual Child Offenders

### 3.1. Prevalence of Female Child Sexual Offenders

Due to different methodologies and samples, prevalence reports of sexual child abuse committed by women vary within the literature. There are two main sources of information for estimating the prevalence of FCSO: Firstly, official reports (i.e., from police or court offices); secondly, victim reports. An overview of the results of different studies and reviews is shown in Table 1 and Table 2.

The comparison of prevalence rates based on official reports (Table 1) and those based on victimization surveys (Table 2) clearly demonstrate a great gap. Sexual offences against children committed by women appear to be underreported and not prosecuted adequately. Table 2 only includes studies with large sample sizes and/or those examined representative samples published from 2000 and onwards. Taking into account earlier studies on smaller and/or clinical samples, even higher prevalence rates for FCSO with male victims are reported. For instance, Fromuth and Buckhart [32] investigated male students from a midwestern (*n* = 253) and a southwestern (*n* = 329) American university. Thirty-eight males from the midwestern university reported that they were sexually abused as a child and 78% furthermore specified a female perpetrator. Forty-three males from the southwestern university had been sexually abused as children, of whom 78% reported a female perpetrator [32].

### 3.2. Characteristics of Female Child Sexual Offenders and Their Victims

Research so far indicates that FCSO are a rather heterogeneous population with different features [5,33,34,35]. However, some common characteristics of FCSO and their victims were found.

The average age of FCSO seems to range from 26–36 [5]. For instance, Faller [3,36] reported on a sample of 40 FCSO with a mean age of 26.1 years [36] and on another sample of 72 FCSO with a mean age of 28 years [3]; and Nathan and Ward [37] reported on 12 FCSO with a mean age of 30 years. The majority of FCSO in empirical research showed a rather low socioeconomic status [5,12,38] with little vocational qualifications [12,39,40]. According to Berner, Briken, and Hill [41], more than 50% of FCSO had experienced sexual and/or physical abuse themselves [41]. Indeed, many studies demonstrated FCSO as being mentally, sexually, and/or physically abused during childhood [12,38,42,43,44,45]. They often show mental health problems, particularly substance abuse [45], personality disorders (passive and/or dependent) with rather low self-esteem [46], and are frequently involved in abusive relationships during adulthood [38,47] or have an absence of intimate relationships [45]. FCSO further appear to be impulsive with low levels of emotional self-regulation [48].

Typically, FSCOs find their victims in their closer social circle [3,16,35,42,49,50]. Often they are their victims’ caregivers, i.e., mothers, other relatives, or babysitters [3,16,38]. The prevalence rates shown in Table 2 indicate that FCSO appear to sexual abuse male victims more often than female victims. However, research results so far are not sufficiently reliably to predict who may be at higher risk to be abused by an adult woman: boys or girls [5]. Victims’ age ranges from infants to adolescents [51,52].

### 3.3. Perception and Handling of Female Child Sexual Offenders

The discrepancy between official reports and victimization surveys on the prevalence of FCSO clearly demonstrates the under-recognition of women who behave in a sexually abusive manner. Official statistics only reflect those women who have had contact with the criminal justice or social service system. This indicates that reporting FCSO to the police or child welfare agencies seems to be a great obstacle. In fact, from the very beginning of scientific confrontation with FCSO in the 1930 [53], women who sexually abuse children have been a powerful social taboo [18]. Women are usually portrayed as victims and as being passive, innocent, and sexually submissive. Moreover, they are primarily normalized as the gatekeepers of sexuality [18]. In terms of anatomy, some have argued that women are receivers of sexuality which might make it difficult to imagine a woman as someone who sexually abuses others [54]. Instead, women are frequently seen as nurturers and protectors in positions of trust. They are thought of as mothers and those who provide care for others. Women who sexually abuse children undermine such normative labels and challenge traditional gender stereotypes that are firmly established in society [18].

#### 3.3.1. Society

The way in which members of a society perceive and respond to certain events is significantly shaped by medial reports [55]. Research so far has shown that media’s representation of sexual offenders is biased [56]. In an analysis of 29 newspaper articles published in Australian dailies, Landor and Eisenchlas [56] showed that male sexual offenders are strongly criticized in media reports, whereas female sexual offenders are usually described in a more sympathetic way. Furthermore, the articles on FCSO usually contain excuses to justify or lessen the seriousness of the women’s abusive behavior [56]. Hayes and Baker [18] also analyzed the way in which the media reports on women who sexually abused children. The authors theorized that media reports tend to reinforce traditional gender stereotypes and therefore suppress the development of a public awareness of sexual offences committed by women. Examining 487 media reports from Australia and the United Kingdom, they found that the media mainly presents FCSO as aberrations and pariahs (in terms of outcasts), and thus do not contribute to an atmosphere supporting the safe and timely reporting of offences by victims [18].

Mackelprang and Becker [57] demonstrated that this unequal perception of men and women who sexually offend against children is in fact reflected in societal judgements. The authors asked 432 undergraduate students to judge teacher sexual offence vignettes (e.g., amount of time the offender should be incarcerated) that varied by offender’s gender and attractiveness. For all outcome measures reflecting punitive judgements and attitudes towards the offender, female teachers who had had a sexual relationship with a student were evaluated more leniently and judged less punitively than male teachers who did the same. In addition, there has been an even greater tolerance for FCSO when they were described as attractive instead of unattractive. This effect was not observed for the vignettes on male child sexual offenders [57].

#### 3.3.2. Professionals

Professionals in healthcare, criminal justice, and child protection systems were also shown to respond inappropriately in cases of child sexual abuse committed by women [58,59,60,61,62]. For instance, children’s disclosure was brushed aside as fantasies [63] or abusive women gained further access to potential victims [64]. In 2010, Mellor and Deering [20] examined professional responses and attitudes toward FCSO. A total of 231 Australian psychiatrists, psychologists, probationary psychologists, and child protection workers were presented with a variation of vignettes describing women and men who had sexually offended against children. Afterwards they completed a questionnaire on their attitudes to women’s offending behavior toward children. Compared to male-perpetrated child sexual abuse, female-perpetrated child sexual abuse was more likely to be rated leniently. This “indicates that a level of professional minimization towards female-perpetrated child sexual abuse exists” [20] (p. 433). Psychotherapists who treat young patients experiencing mother-incest-abuse initially often struggle with the idea of reporting these cases [65]. As Haliburn concluded, the frequency of mental health patients reporting histories of child sexual abuse does not surprise clinicians anymore. However, when the perpetrator is a woman, clinicians’ reaction often is “shock and disbelief and a tendency to be dismissive” [65] (p. 423).

#### 3.3.3. Victims and Offenders

As a consequence of FCSO being a social taboo, their victims often have difficulties in recognizing their experiences as sexually abusive [66] and feel intensly confused [67]. It is not unusual that FCSO disguise their abusive behavior as part of childlare activities [67]. This might in part be the reason why in fact even the offenders themselves have difficulties in recognizing their behavior as sexually abusive [68]. FCSO’ victims are faced with serious issues regarding the disclosure of their abuse [69], thus hesitating more often to disclose the abuse than victims of male offenders [70]. It is particularly worth mentioning that victims of FCSO in early treatment stages even appear to lie to their therapists about their abuser’s sex, claiming that they were perpetrated by a man [71]. These difficulties might be even worse when the female perpetrator is the own mother [72]. Usually shrouded in secrecy, Haliburn [65] called mother–child incest a “double betrayal”, since both, the violation of trust as well as the exploitation of the child’s affection and dependency needs to take place. Individuals who were sexually abused by their own mother were described as feeling additional shame and stigma [73].

## 4. Implications

Based on the outlined research, we propose some general implications for professionals in health care followed by more specific recommendations for researchers in the field of sexual health. As there are many possible clinical and research implications, we do not make any claim to comprehensiveness.

### 4.1. General Implications

Offences of FCSO are underreported and therefore FCSO are difficult to study. The resulting knowledge gap about FSCOs reduces the quality of child protection and treatment services. We therefore deem it particularly necessary for health care professionals to overcome the social taboo that is FCSO.

As mentioned, there seems to be a marked resistance in the general public and the health care system to detect FCSO [19]. Historically, the same kind of resistance was documented for the acceptance and awareness of men who sexually abuse children [74]. Thus, in accordance with Mellor and Deering [20], we state that the overall awareness and appropriate attitude towards FCSO have to be improved in health care, criminal justice, and child protection systems. Since a structured training as proposed by Mellor and Deering [20] may strain the organizational capacities of most institutions, we recommend an increased engagement of the issues concerning FCSO in internal conferences and discussions. This may lead to a more open discussion of FCSO among colleagues and therefore to a stronger representation of the issue in the professional’s mind. Consequently, this should help to uncover the abusive behavior for both victims and offenders.

As media portrayals of FCSO and their victims are generally inadequate [18], instructions for journalists concerning the appropriate attitude towards FCSO are also deemed necessary.

The tendency to deny and minimize, leads to FCSO being a hidden phenomenon, undeniably difficult to uncover (cf. References [18,75,76,77,78,79,80]). We therefore advise professionals in both clinical practice and scientific research to consciously challenge and control their own underlying mechanisms of denial when confronted with cases of FCSO.

Since it is assumed that victims of FCSO and even FCSO themselves have difficulties to recognize the women’s behavior as sexually abusive [18], we deem an active approach towards FCSO in order to meet their needs is most appropriate. Therefore, we propose education and information within health care, justice, and other systems. For instance, wherever undetected FCSO and/or their victims might occur (e.g., pediatrician practices, youth welfare offices, kindergarten, schools, counselling for victims of sexual offending, women’s house), an educational brochure could be distributed to adults. It might briefly and simply inform the reader about the fact that women are also capable of sexually abusing children including contact details for both FCSO and victims. By this, a network including members of different professions within health care, justice, and other systems might be built so that regular communication and information between different systems regarding the issues of FCSO can be established.

Furthermore, we find it important that FCSO are also recognized by the general public, making public outreach necessary. Where great campaigns and activities for public outreach are difficult to implement, we suggest rather simple ways to contribute to public awareness of FCSO. Media reports can be considered to have an impact on social discourses [81] and to play a crucial role in the way society perceives and responds to women who sexually abuse children and therefore undermine traditional gender stereotypes [55]. Professionals in the health care system are sometimes being consulted as experts for child sexual abuse, child sexual offenders, or any other related topic for newspaper articles or television reports. In these situations, we believe in the professional’s responsibility to address child sexual abuse by women as an existing problem which can be just as harmful for the victims as child sexual abuse by men can be. In time, the topic may affect and receive more attention in broader circles of the general public and be discussed beyond the professional fields.

### 4.2. Implications for Researchers

FCSO are usually only investigated when they are registered in the judicial system (i.e., when they were reported to the police by victims or others). As described earlier, women who sexually offended against children remain undetected very often due to several reasons [70]. We therefore encourage researchers to attempt additional and more active approaches to recruit FCSO for their examinations. For instance, as research results indicate that FCSO are young women between mid-twenties and mid-thirties, online surveys may be an appropriate tool to investigate this population. Online surveys are a highly economic way to reach out for participants who are inhibited due to several barriers such as women who sexually abuse children and furthermore provide a high level of identity protection. Both of which should be helpful when trying to recruit FCSO. Additionally, online surveys are highly suitable to reach women who are at risk to sexually abuse children but did not yet offend against a child. Besides, researchers already investigated female sexual offenders on the internet concluding that they use the internet to connect with like-minded women [82,83].

When creating the survey, we recommend simple language due to the relatively low socioeconomic status of FCSO [5]. As many FCSO reported on being abused in their childhood [41] and having mental health problems, such as depression [84] and alcohol abuse [45], researchers are advised to distribute their study link in internet forums and self-help groups on the internet for victims of child abuse, depressive patients and alcohol abusers.

Additionally, we suggest that researchers should not only include FCSO in their online surveys but also those women who are solely at risk to offend against children and did not yet offend against children. Differentiation between women who have a sexual interest in children can be made, e.g., those who have a pedophilic interest as motive vs. those who have other motives for offending against children or those who are willing to be in treatment vs. those who do not want to be in treatment. These differentiations may lead to different subgroups with varying characteristics implying different research questions and assumptions. This would be in alignment with research on men who are sexually interested in children [85,86,87].

Finally, if the conditions regarding institutional capacity and financial management are met, qualitative interviews with FCSO or those women who are at risk to sexually offend against children would be valuable to ascertain more details of FCSO’ characteristics, their offence behavior, and their specific underlying mechanisms of denial and minimization.

## 5. Conclusions

General public and professionals both reinforce and maintain traditional gender stereotypes which appear to be barriers to the detection of FCSO [80]. The “culture of denial” surrounding women who are sexually offensive [67] conceals their acts as “silent crimes” [88]. It is likely that the diverting prevalence rates based on different sources (official reports vs. victimization surveys) are related to this biased perception and inappropriate handling of FCSO. As a result, FCSO are underreported and difficult to study which leads to insufficient scientific knowledge. The lack of research data on FSCOs lowers the quality of child protection and treatment services. The fact that even professionals in the judicial and health system appear to be part of this collective repression clearly demonstrates that there is a particular responsibility for researchers and clinicians in the field of sexual health to be aware of their own underlying mechanisms and inner processes of denial. It is important to pursue an active approach towards FCSO. Overcoming the social taboo of FCSO is obligatory, especially in the light of the harsh consequences for victims of FCSO [89]. Moving beyond traditional gender stereotypes seems to be necessary to get over the confusion that women considered so far as caregivers, guardians, and defenders (cf. Reference [90]) are able to be just as sexually abusive to children as men.

## Figures and Tables

**Table 1:** Prevalence rates (PR) for female child sexual offenders (FCSO) based on official reports.

Reference	Country	Year	Source of Information	Sample Size (Offenders)	PR for FCSO (%)
[21]	Australia	2005	Incidents reported in official statistics	1,294,000	1.7 ^a^
[22]	Canada	2017	Accusations reported in official crime statistics	4703	3.7
[23]	USA	1991–1996	National Incident-Based Reporting System (incidents reported to law enforcement)	8539 (victims younger age 6)	12
				12,260 (victims aged 6–12)	6
				20,005 (victims aged 12–17)	3
[24]	Germany	2007–2014	Convictions reported in official crime statistics	14,069	1.4
[24]	Germany	2016	Inmates remanded in custody reported in official crime statistics	402	1.7

Studies included all met the definition of child sexual abuse as experiencing vaginal/anal penetration or attempted penetration with fingers, penis, objects and/or oral sex, attempted oral sex, unwanted sexual touching or fondling or any other kind of sexual interaction before the age of 17. ^a^ Only includes female relatives, no female strangers.

**Table 2:** Prevalence rates (PR) for female child sexual offenders (FCSO) based on victimization surveys.

Reference	Country	Year	Source of Information	Sample Size (Victims)	PR for FCSO (%)
[25]	USA	1996	National Incidence Study by the National Center on Child Abuse and Neglect (NCCAN)	300,200	12
[26]	USA	2009/10	Child protective system reports from the National Child Abuse and Neglect Data System (NCANDS)	66,765	20
[27]	UK	2008/09	Analyses of call records from ChildLine (free hotline for children in need)	12,268	17
[28]	UK	2005/06	Analyses of call records from ChildLine	6763 ^a^	44 ^b^ (male victims)
					5 (female victims)
[29]	USA	1995–1997	Cohort study among adult members of the Kaiser Permanent’s Health Appraisal Centre in San Diego	1276 (male victims)	20.8
				2310 (female victims)	2.1
[30]	UK	2007/08	Analyses of call records from ChildLine	1803 (male victims)	26^c^
[15]	Ireland	2001	Retrospective data on adult accounts of childhood abuse from SAVI (population-based interview survey)	270 (male victims)	14.8
				407 (female victims)	1.7
[31]	Germany	2011	Retrospective data from a population-based survey on child sexual abuse among 16–40 year olds	83 (male victims)	15.3
				404 (female victims)	1.5

Studies included all met the definition of child sexual abuse as experiencing vaginal/anal penetration or attempted penetration with fingers, penis, objects and/or oral sex, attempted oral sex, unwanted sexually touching or foundling or any other. ^a^ To our knowledge, the data on ChildLine cited by Roberts [28] have not been published entirely elsewhere. Due to this, it was not possible to specify the sample size by gender. ^b^ PR specified by gender indicates the proportion of female perpetrators within gender groups. For instance, a PR of 44 for male victims indicates that 44% of all male victims reported a female perpetrator. ^c^ Only includes female relatives, no female strangers.
